# Caste-specific storage of dopamine-related substances in the brains of four *Polistes* paper wasp species

**DOI:** 10.1371/journal.pone.0280881

**Published:** 2023-01-26

**Authors:** Ken Sasaki, Hideto Yoshimura, Masakazu Nishimura

**Affiliations:** 1 Graduate School of Agriculture, Tamagawa University, Machida, Tokyo, Japan; 2 Honeybee Science Research Center, Tamagawa University, Machida, Tokyo, Japan; 3 Division of Agro-Environment Research, Tohoku Agricultural Research Center, NARO, Morioka, Iwate, Japan; Albert-Ludwigs-Universitat Freiburg, GERMANY

## Abstract

How the role of dopamine differs according to the evolution of eusociality and how it is required in the flexible society of *Polistes* paper wasps need further clarification. In the present study, we compared the storage and usage of dopamine-related substances in brains between the castes of paper wasps. The head widths, lipid stores in the abdomen, and levels of biogenic amines in the brains were measured in newly emerged females before male emergence (workers) and after male emergence (gynes) in four *Polistes* species. The head widths and the lipid stores were significantly larger in gynes than workers in *P*. *snelleni*, *P*. *rothneyi*, and *P*. *jokahamae*, whereas they did not differ between castes in *P*. *chinensis*. The levels of dopamine precursors in the brains were significantly higher in gynes than workers in *P*. *snelleni*, *P*. *chinensis*, and *P*. *rothneyi*, whereas those of dopamine and its metabolites did not differ between castes in these species. In *P*. *jokahamae*, the levels of dopamine precursors and dopamine in the brains did not differ between castes, but those of a dopamine metabolite were significantly higher in gynes than workers. Thus, the caste differences in the levels of dopamine-related substances did not always match body sizes and nutritional reserves. Foundresses in *P*. *rothneyi* had significantly lower levels of dopamine precursors and higher levels of dopamine and its metabolite than newly emerged gynes. These results suggested that in several *Polistes* species, dopamine precursors were stored in the brain without dopamine biosynthesis at emergence, and then converted into dopamine in foundresses during colony founding. These neuroendocrinal states in *Polistes* species largely differed from those in eusocial bees.

## Introduction

Eusociality has evolved independently among a wide range of insect taxa [1,2]. Eusociality in Hymenoptera is a typical case of convergent evolution between different groups, including wasps, ants, and bees [3–6]. In these eusocial species, females differentiate into reproductive (queen or gyne) or non-reproductive (worker) castes morphologically or behaviorally. In eusocial hymenopterans with dependent colony founding by swarming, where a queen is accompanied by workers, such as in honey bees, caste differentiation involves morphological specialization. In temperate eusocial paper wasps and bumble bees with independent colony founding, where a hibernated gyne initiates nest founding without workers, larger body sizes are observed in gynes than in workers due to nutritional reserves for hibernation. However, the external morphological characteristics of workers are preserved in gynes. This is because the foundress performs worker-like behaviors (e.g., nest construction, brood caring, and foraging) during a particular colony founding stage. Thus, the degree of morphological caste differentiation depends on the manner of colony founding and behavioral specialization, which may be related to the specialization of physiological state in each caste. Caste differences in physiological states in the brain provide clues for understanding the evolution of the reproductive division of labor, however, comparative studies focusing on neuroactive substances are limited.

Nutrition during the larval stage is an important factor affecting caste differentiation and influences external morphology, body size, lipid storage, and brain physiology at the adult stage [7–9]. The caste dimorphism of brain physiology, such as biogenic amine levels, is initially formed at the emergence of adults. Biogenic amines are neuroactive substances that have multiple functions such as neurotransmitters, neuromodulators and neurohormones in insects [10–12]. In honey bees and bumble bees, the levels of dopamine, a biogenic amine, in the brains are significantly higher in gynes than workers [13,14]. In the honey bee, the differences are caused by intakes of a dopamine precursor tyrosine from royal jelly during the larval stage [15] and the expression of enzyme genes involved in dopamine biosynthesis during the pupal stage [16]. A greater amount of dopamine in the brains of honey bee gynes can contribute to fighting behavior between virgin gynes within a nest [15,17] and mating flight activity [18,19]. Dopamine also has a hormonal function in ovarian activation through dopamine receptors expressed on the ovaries and fat bodies in several eusocial hymenopterans [20–23]. The multiple functions of dopamine may be inherited from the ancestral solitary species and shared in eusocial Hymenoptera [12,23]. However, other biogenic amines except for dopamine have similar reproductive functions in females of solitary Hymenoptera [24]. Therefore, investigations of dopamine usage in the reproductive castes among different groups of eusocial hymenopterans with different levels of caste differentiation are required.

Temperate *Polistes* wasps form a large eusocial group in Hymenoptera and have large variations in caste determination. The caste in temperate *Polistes* wasps is initially influenced by nutrition, vibration stimuli, and photoperiod in preimaginal stages [8,25–31]. The caste in several *Polistes* species is finally determined by external environmental stimuli, including photoperiod, temperature, and colony conditions at the adult stage [8,31–35], with influences of caste-related physiology during preimaginal stages. Therefore, the brain physiology in temperate *Polistes* species may have high plasticity at emergence to change the caste in response to environmental factors during the adult stage. However, this possibility has not been evaluated.

In the present study, we compared the brain levels of biogenic amines, especially dopamine-related substances, between newly emerged workers and gynes in four species of temperate *Polistes* paper wasps. The temperate *Polistes* species are an ideal model for adaptive physiological states in the brains between castes, because of different levels of caste differentiation among species [6,8,36]. In such species, intermediate or undifferentiated characteristics in the brains between castes are expected, in comparison to the honey bee with clear caste dimorphism of brain characteristics. Therefore, elucidation of dopamine function in the primitive eusocial *Polistes* species may contribute to our understanding of the common physiological bases underlying behavioral caste differentiation among different hymenopteran groups with convergently evolved eusociality. Because newly emerged females have a brain at the initial neuroendocrinal state without social behavioral experience, we investigated caste differences at the initial neuroendocrinal state and tested the possibility of high plasticity in the brain physiology of temperate *Polistes* wasps. In addition, since neural development of the brain occurs early in adult stage in paper wasps [37,38], the initial neuroendocrinal state at emergence may change thereafter. Therefore, we compared the levels of dopamine-related substances between newly emerged gynes and foundresses in a *Polistes* species to discuss the usage of dopamine at different stages of adult life in gynes.

## Materials and methods

### Collection of the nests and sampling conditions

Nests of four species of temperate paper wasps, *P*. *snelleni*, *P*. *chinensis*, *P*. *rothneyi*, and *P*. *jokahamae*, were collected from the field in Nagano prefecture, Iwate prefecture, and Tokyo in Japan. In these species, a single foundress usually founds the nests from April to May, and the nests produce workers until August. Multi-foundress nest founding is at a low rate in these species in Japan [39,40]. Then, males and gynes emerge and disperse from the nests. After mating, the mated gynes hibernate during winter. The four *Polistes* species have different characteristics including worker reproductive ability and plasticity of caste-fate determination during adult stage. In *P*. *chinensis*, workers have developed ovaries under queenright conditions [41–43] and contribute a part of male production [44]. This characteristic is different from the other three species, and eusociality in *P*. *chinensis* is more primitive than in the others. In *P*. *jokahamae*, adult females can activate ovaries under queenless long-day conditions as egg-laying workers, whereas they can store the lipid in the abdomen without ovarian activation under queenless short-day conditions for hibernation [31]. The photoperiod-dependent caste-fate determination in adults has been reported in *P*. *jokahamae* among the four species.

To obtain newly emerged workers and gynes, we collected the nests containing broods and adult workers with or without adult males and kept the nests in the laboratory. Although the timing of male and gyne emergence is slightly different between the *Polistes* species [45], in general, females emerging before or after male emergence correspond to workers or gynes, respectively [46]. Therefore, females that emerged from the nests without any adult males were defined as workers, whereas females that emerged from the nests with several adult males were defined as gynes. Newly emerged females (emergence from cocoon within 24 h) were euthanized by liquid nitrogen and used to measure the head width and lipid stores in the abdomen, or the biogenic amine levels in the brain. For the measurements of the head widths and lipid stores, emerged females were euthanized and stored in the freezer at -20°C. For the measurement of biogenic amines in the brain, emerged females were euthanized by liquid nitrogen and kept in a cryogenic container (DR3, Taiyo Nippon Sanso, Tokyo, Japan) filled with liquid nitrogen until quantification to prevent the degradation of biogenic amines.

To compare the biogenic amine levels between newly emerged gynes and foundresses, after hibernation the foundresses of *P*. *rothneyi* were collected in mid-April. This species possesses standard characteristics of eusocial paper wasps such as pre-imaginal caste determination and infertile workers under queenright condition, and therefore, this species is suitable for determining typical changes in neuroendocrinal states from emergence to nest founding in gynes. Six of the 10 foundresses were collected from the founding nests by a single foundress with eggs but no larvae, pupae, and adult workers. The other four females were collected on the same day as the foundresses, but not from the nests. These females peeled the bark of the wood for nest materials and had matured eggs in their ovarioles as in foundresses. Therefore, they were treated as foundresses. All foundresses were euthanized by liquid nitrogen and kept in liquid nitrogen until quantification.

### Measurements of head width and lipid stores in the abdomen

To determine the differences in external and internal characteristics between the castes, the head width and the lipid stores in the abdomen were measured as body size and nutritional reserves, respectively. The widest part of the head of newly emerged females was measured using a caliper with a precision of 0.01 mm. To measure the lipid stores in the gaster including the ovary, abdomens were placed in a 1.5 mL microtube, dried at 50°C for 3 days, and weighed on a balance. After weighing, the abdomens were extracted in diethyl ether for 24 h, washed in fresh diethyl ether, and dried for another 3 days at 50°C [31,47]. Following the second drying, abdomens were weighed again. Fat content was calculated from the difference between the dry mass and the dry fatless mass post-extraction. Dry mass was measured by an electronic balance with a precision of 0.0001 g.

### Measurements of biogenic amines in the brain

To compare the physiological state of each caste, and to detect the storage and usage of dopamine-related substances during colony founding in the foundress, biogenic amines in brains were quantified. Frozen brains were dissected and treated for analyses by high-performance liquid chromatography with electrochemical detection (HPLC-ECD), following the method by Sasaki *et al*. [14]. Frozen brains were dissected in ice-cold honey bee saline (128.33 mM NaCl, 2.68 mM KCl, 1.80 mM CaCl_2_, pH 6.7) on a Peltier cooling unit (Kenis Ltd, Osaka, Japan) at approximately 4°C under a dissecting microscope. A single dissected brain, including mushroom bodies, antennal lobes, and optic lobes with subesophageal ganglion, was homogenized with a microglass homogenizer in 100 μL of ice-cold 0.1 M perchloric acid containing 0.1 ng/μL 3,4-dihydroxybenzylamine for 2 min. Each sample was then transferred into a 1.5 mL microtube and centrifuged at 15,000 × *g* for 30 min at 4°C. Each supernatant was transferred into two microvials for analysis by HPLC-ECD. Two HPLC-ECD systems were used to measure the level of biogenic amines in the brain and to obtain appropriate retention times of the peaks of tyrosine and L-DOPA in the chromatogram. One HPLC-ECD system was used for dopamine precursors (tyrosine and 3,4-dihydroxyphenylalanine: L-DOPA) and comprised a solvent delivery pump (PU-4580, JASCO, Tokyo, Japan), a refrigerated automatic injector (AS-4550, JASCO), and a C18 reversed-phase column (250 mm × 4.6 mm id., 5-μm average particle size, MG, Osaka Soda, Osaka, Japan) maintained at 35°C. An electrochemical detector (ECD-700, EICOM, Osaka, Japan) set at 0.8–0.85 V was used under 35°C. The mobile phase contained 83 mM citric acid monohydrate, 17 mM sodium acetate, 13 μM 2Na-EDTA, and 2.3 mM sodium-1-octanesulfonate. Into this solution, 7% methanol was added. The flow rate was kept constant at 0.7 mL/min.

The measurements of dopamine, *N*-acetyldopamine, tyramine, and serotonin were made using another HPLC-ECD system and comprised a solvent delivery pump (PU-4180, JASCO), a refrigerated automatic injector (AS-4050, JASCO), and a C18 reversed-phase column (250 mm × 4.6 mm id., 5-μm average particle size, UG120, Osaka Soda) maintained at 35°C. An electrochemical detector (ECD-700, EICOM) set at 0.8–0.85 V was used under 35°C. The mobile phase contained 0.18 M monochloroacetic acid and 40 μM 2Na-EDTA, which was adjusted to pH 3.6 with NaOH. Into this solution, 1.62 mM sodium-1-octanesulfonate and 5% CH₃CN were added. The flow rate was kept constant at 0.7 mL/min.

In both HPLC-ECD systems, external standards were run before and after the sample runs for the identification and quantification of functional monoamines (dopamine, tyramine, and serotonin), dopamine precursors (tyrosine and L-DOPA), and a dopamine metabolite (*N*-acetyldopamine). The peaks of these substances were identified by comparing both the retention time and the hydrodynamic voltammograms with those of the standard. Measurements based on the peak area of the chromatograms were obtained by calculating the ratio of the peak area of a substance to the peak area of the standards.

To normalize the brain levels of biogenic amines based on protein content, proteins were quantified by the Bradford method [48]. Precipitated protein pellets were neutralized with 50 μL 0.5 M NaOH following biogenic amine extraction. After the ultrasonic dissolution of the pellet for 15 min, the solution was diluted with 200 μL 0.1 M phosphate buffer (pH 7.0). As a standard solution, 5 mg bovine serum albumin was dissolved in 0.5 M NaOH with 0.1 M phosphate buffer (1:4, 5 mg/mL) and diluted with the same buffer 1/10, 1/20, 1/40, and 1/80-fold. The samples and standards were reacted using protein assay dye reagent (500–0006, Bio-Rad, Hercules, CA, USA) in a 96 well-plate and mixed for 5 min. The absorbance of the mixtures was measured by using a microplate reader (MRP-A100, AS ONE, Osaka, Japan) with a 590-nm wavelength. Protein concentrations in the brains were determined from the calibration curve of the standard solutions.

### Statistics

Because the data of several groups did not fit a normal distribution and had different variances (S6 Table), non-parametric tests were used for the statistical analyses. The head widths, the lipid stores in the abdomen, the ratios of lipid stores to head width, and the biogenic amine levels in the brains were compared between the newly emerged workers and gynes by the Mann–Whitney U-test (significant differences were considered at *p* < 0.05). The biogenic amine levels were also compared between the emerged gynes and foundresses by the Mann–Whitney U-test. The correlations between the head widths and lipid stores were examined by the Spearman’s rank correlation test. The statistical analyses were performed using computer programs in R (version 3.6.0) and StatView (version 5.0, SAS). To indicate a phylogenetic relationship among the four species of paper wasps [49], the honey bee (*Apis mellifera*), and the bumble bee (*Bombus ignitus*), and to discuss the phylogenetic constraints among these species, a phylogenetic tree was made by using Molecular Evolutionary Genetics Analysis (MEGA version 11.0.10), based on the DNA sequence of 16S rRNA. The DNA sequences of 16S rRNA in the species were obtained from the database with accession numbers AB284542.1 for *P*. *snelleni*, AB284537.1 for *P*. *chinensis*, AB284540.1 for *P*. *rothneyi*, AB284539.1 for *P*. *jokahamae*, DQ788031.1 for *B*. *ignitus*, AP018435.1 for *A*. *mellifera*, and EU746617.1 for *Nasonia vitripennis* as the outgroup.

## Results

### Differences in body size and nutritional reserves between castes

**Fig 1:**
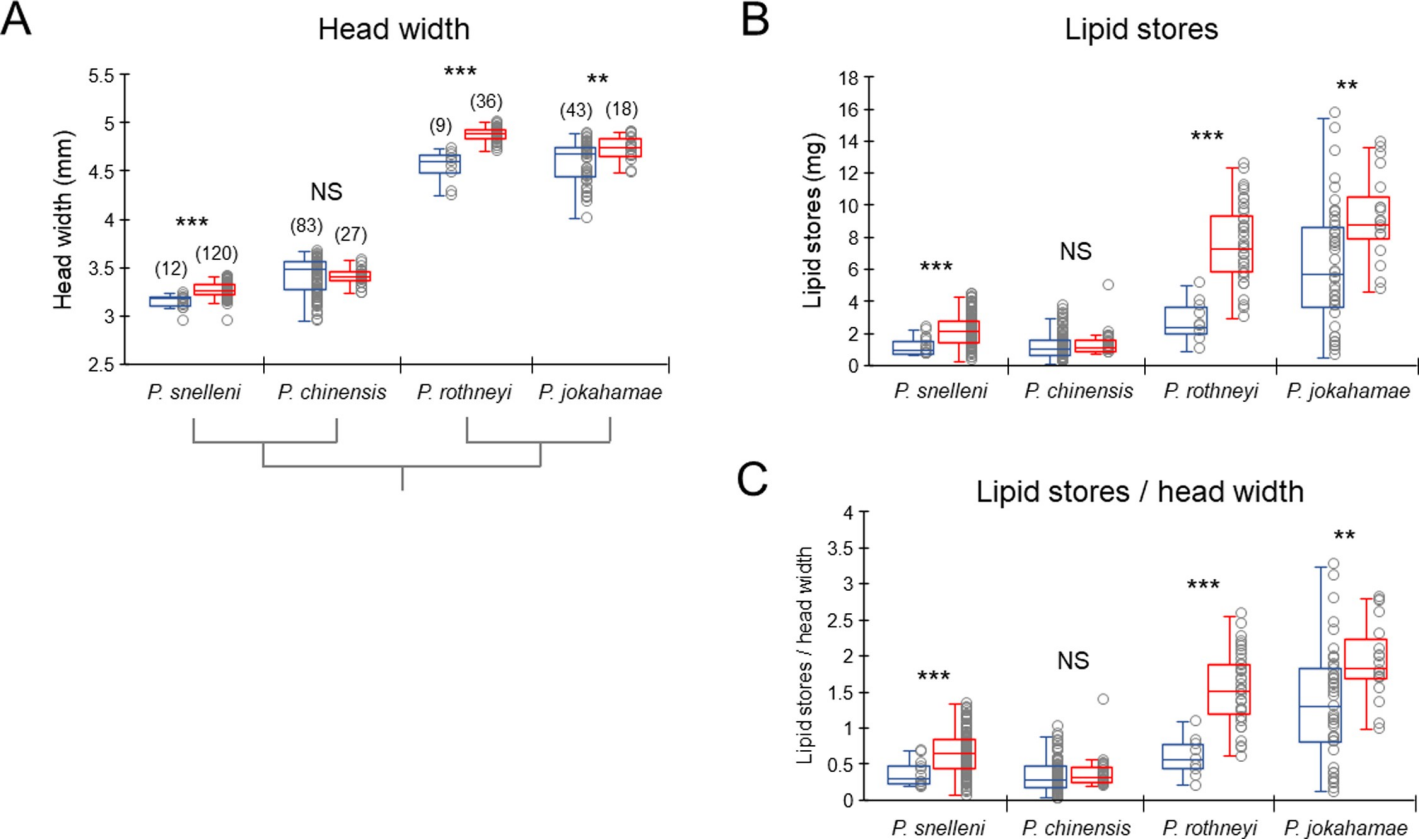
Head width, lipid stores in the abdomen and standardized lipid stores of newly emerged workers and gynes in four species of *Polistes* paper wasps. (A) Head width, (B) lipid stores in the abdomen and (C) ratios of lipid stores to head widths. Blue and red box plots indicate workers and gynes, respectively. Numbers in parentheses in the graph indicate the number of samples examined. The number of samples examined for the lipid stores and standardized lipid stores is the same as those for head width. Asterisks in the graph indicate significant differences between workers and gynes (**: P < 0.01, ***: P < 0.001, NS: not significant, P > 0.05). The phylogenetic relationship is indicated below the X-axis in (A).

Head widths and lipid stores in the abdomen were measured to assess body size and nutritional reserves and compared between castes. The head widths in *P*. *snelleni*, *P*. *rothneyi*, and *P*. *jokahamae* were significantly larger in gynes than in workers, whereas those in *P*. *chinensis* did not differ between castes (Mann–Whitney U-test, *P*. *snelleni*: *z* = -4.615, *p* < 0.001; *P*. *rothneyi*: *z* = -4.554, *p* < 0.001; *P*. *jokahamae*: *z* = -2.585, *p* < 0.01; *P*. *chinensis*: *z* = -1.278, *p* = 0.201; Fig 1A). The lipid stores in the abdomen in *P*. *snelleni*, *P*. *rothneyi*, and *P*. *jokahamae* were significantly larger in gynes than in workers, whereas those in *P*. *chinensis* did not differ between castes (U-test, *P*. *snelleni*: *z* = -3.522, *p* < 0.001; *P*. *rothneyi*: *z* = -4.228, *p* < 0.001; *P*. *jokahamae*: *z* = -2.973, *p* < 0.01; *P*. *chinensis*: *z* = -1.229, *p* = 0.219; Fig 1B). The head widths were positively correlated with the lipid stores in the four species (Spearman’s rank correlation test, *P*. *snelleni*: *r*_*s*_ = 0.302, *p* < 0.001, *n* = 132; *P*. *chinensis*: *r*_*s*_ = 0.583, *p* < 0.001, *n* = 110; *P*. *rothneyi*: *r*_*s*_ = 0.653, *p* < 0.001, *n* = 45; *P*. *jokahamae*: *r*_*s*_ = 0.596, *p* < 0.001, *n* = 61). To standardize the lipid stores by body size, the ratios of lipid stores to head widths were calculated. The ratios of lipid stores to head widths in *P*. *snelleni*, *P*. *rothneyi*, and *P*. *jokahamae* were significantly larger in gynes than in workers, whereas those in *P*. *chinensis* did not differ between castes (U-test, *P*. *snelleni*: *z* = -3.38, *p* < 0.001; *P*. *rothneyi*: *z* = -4.2, *p* < 0.001; *P*. *jokahamae*: *z* = -2.91, *p* < 0.01; *P*. *chinensis*: *z* = -1.354, *p* = 0.176; Fig 1C). Thus, the head widths, lipid stores and lipid stores standardized by head widths differed between castes in *P*. *snelleni*, *P*. *rothneyi*, and *P*. *jokahamae*, but not in *P*. *chinensis*.

### Differences in biogenic amine levels in the brains between castes

**Fig 2:**
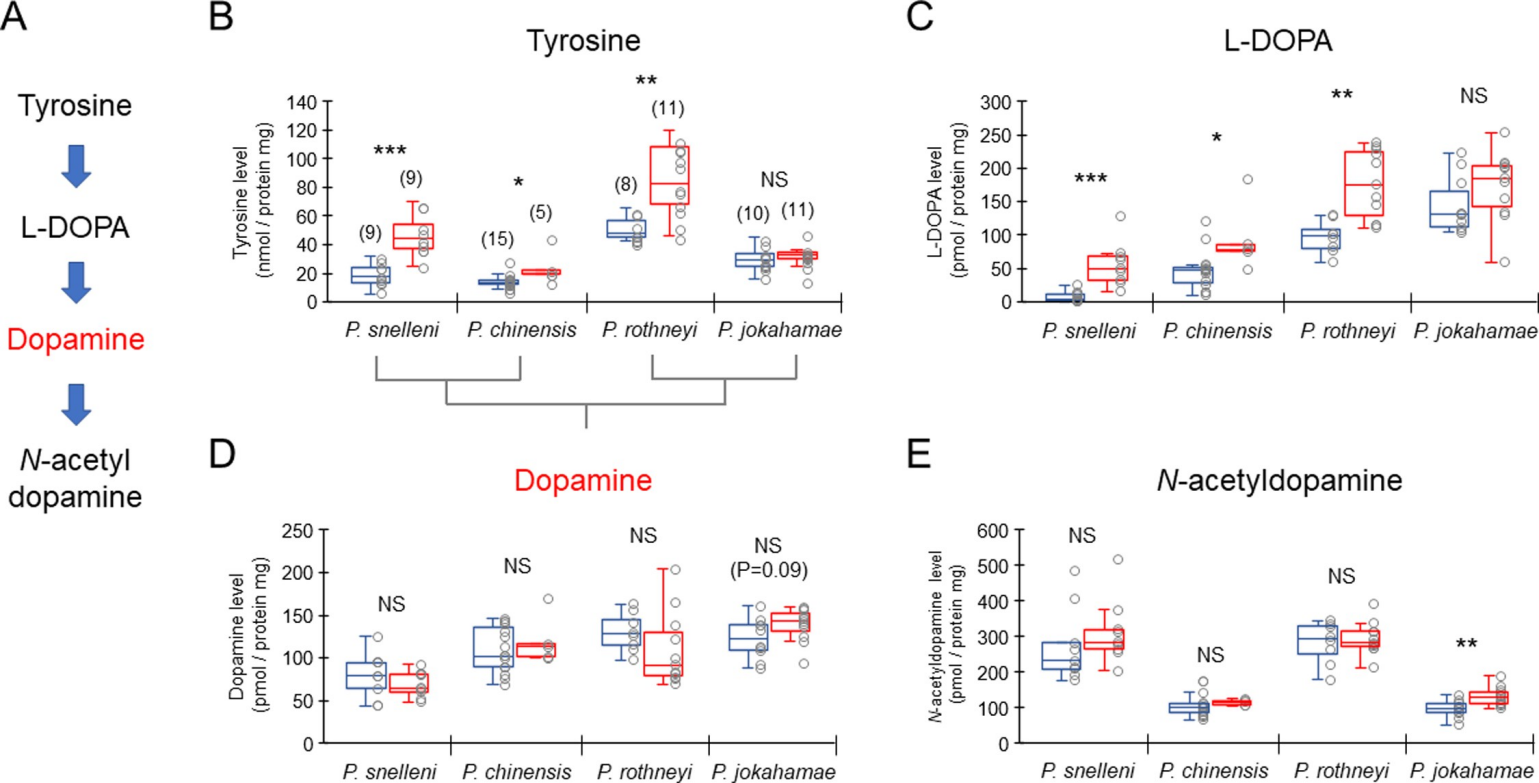
Amounts of dopamine-related substances in the brains of newly emerged workers and gynes in four species of *Polistes* paper wasps. (A) Dopamine synthetic pathway, (B) tyrosine levels, (C) L-DOPA levels, (D) dopamine levels, and (E) *N*-acetyldopamine levels. Blue and red box plots indicate workers and gynes, respectively. Numbers in parentheses in the graph indicate the number of samples examined. The number of samples is the same for four substances. Asterisks in the graph indicate significant differences between workers and gynes (*: P < 0.05, **: P < 0.01, ***: P < 0.001, NS: not significant, P > 0.05). The phylogenetic relationship is indicated below the X-axis in (B).

Tyrosine is a precursor of L-DOPA and dopamine in the brains (Fig 2A), and the levels were significantly higher in gynes than workers in *P*. *snelleni*, *P*. *chinensis*, and *P*. *rothneyi*, but did not differ between castes in *P*. *jokahamae* (Mann–Whitney U-test, *P*. *snelleni*: *z* = -3.400, *p* < 0.001; *P*. *chinensis*: *z* = -2.226, *p* < 0.05; *P*. *rothneyi*: *z* = -2.973, *p* < 0.01; *P*. *jokahamae*: *z* = -0.704, *p* = 0.481; Fig 2B). L-DOPA is also a precursor of dopamine in brains (Fig 2A), and the levels were significantly higher in gynes than workers in *P*. *snelleni*, *P*. *chinensis*, and *P*. *rothneyi*, but did not differ between castes in *P*. *jokahamae* (U–test, *P*. *snelleni*: *z* = -3.488, *p* < 0.001; *P*. *chinensis*: *z* = -2.226, *p* < 0.05; *P*. *rothneyi*: *z* = -3.138, *p* < 0.01; *P*. *jokahamae*: *z* = -1.620, *p* = 0.105; Fig 2C). Dopamine levels in the brains did not significantly differ between castes in all four species (U–test, *P*. *snelleni*: *z* = -0.751, *p* = 0.453; *P*. *chinensis*: *z* = -0.655, *p* = 0.513; *P*. *rothneyi*: *z* = -1.569, *p* = 0.117; Fig 2D), but the dopamine levels of gynes tend to be higher than those of workers in *P*. *jokahamae* (*z* = -1.690, *p* = 0.091). *N*-acetyldopamine is a metabolite of dopamine (Fig 2A), and the levels in the brains did not significantly differ between castes in *P*. *snelleni*, *P*. *chinensis*, and *P*. *rothneyi*, but were significantly greater in gynes than workers in *P*. *jokahamae* (U-test, *P*. *snelleni*: *z* = -1.369, *p* = 0.171; *P*. *chinensis*: *z* = -1.615, *p* = 0.106; *P*. *rothneyi*: *z* = -0.083, *p* = 0.934; *P*. *jokahamae*: *z* = -2.817, *p* < 0.01; Fig 2E).

**Fig 3:**
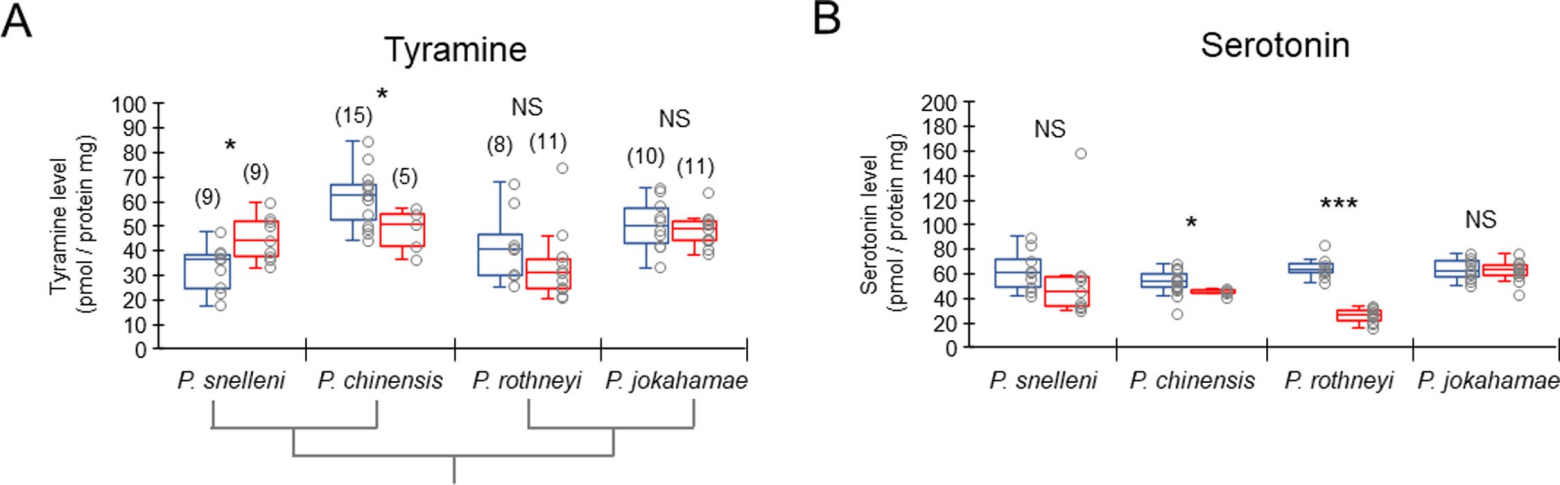
Amounts of tyramine and serotonin in the brains of the workers and gynes of four species of *Polistes* paper wasps. (A) Tyramine levels and (B) serotonin levels. Blue and red box plots indicate workers and gynes, respectively. The number of samples examined for serotonin is the same as those for tyramine. Asterisks in the graph indicate significant differences between workers and gynes (*: P < 0.05, ***: P < 0.001, NS: not significant, P > 0.05). The phylogenetic relationship is indicated below the X-axis in (A).

The levels of tyramine were significantly higher in gynes than workers in *P*. *snelleni* and significantly higher in workers than gynes in *P*. *chinensis*, but did not differ between the castes in *P*. *rothneyi* and *P*. *jokahamae* (U-test, *P*. *snelleni*: *z* = -2.252, *p* < 0.05; *P*. *chinensis*: *z* = -2.139, *p* < 0.05; *P*. *rothneyi*: *z* = -1.404, *p* = 0.160; *P*. *jokahamae*: *z* = -0.704, *p* = 0.481; Fig 3A). The levels of serotonin were significantly higher in workers than gynes in *P*. *chinensis* and *P*. *rothneyi*, but did not differ between castes in *P*. *snelleni* and *P*. *jokahamae* (U-test, *P*. *snelleni*: *z* = -1.634, *p* = 0.102; *P*. *chinensis*: *z* = -2.313, *p* < 0.05; *P*. *rothneyi*: *z* = -3.633, *p* < 0.001; *P*. *jokahamae*: *z* = -0.141, *p* = 0.888; Fig 3B).

### Comparison of biogenic amine levels in the brains between newly emerged gynes and foundresses

To determine whether the levels of biogenic amines change from emergence to the colony founding stage, the levels of dopamine-related substances were quantified in foundresses in *P*. *rothneyi*. The levels of dopamine precursors, tyrosine, and L-DOPA were significantly lower in foundresses than in newly emerged gynes (Table 1). In contrast, the levels of dopamine and *N*-acetyldopamine were significantly higher in foundresses than in newly emerged gynes (Table 1). The ratios of the levels of tyrosine and L-DOPA in foundresses to those in newly emerged gynes were 0.057 and 0.092, respectively, whereas the ratios of dopamine and *N*-acetyldopamine were 5.519 and 1.328, respectively. These values indicated a large conversion to dopamine from precursors in the brains of foundresses.

**Table 1:** Levels of dopamine-related substances and other monoamines in the brains of foundresses (F) and the comparison with those of newly emerged gynes (G) in *Polistes rothneyi*.

	Brain levels* in F		Brain levels* in G		F–G	F / G	F vs. G (U–test)
	mean	SE	mean	SE			
Dopamine-related substances							
Tyrosine	4938.947	484.246	86510.506	7595.929	-81571.558	0.057	P < 0.001
L-DOPA	16.192	4.499	176.581	15.366	-160.389	0.092	P < 0.001
Dopamine	605.364	107.556	109.677	13.081	495.686	5.519	P < 0.001
*N*-acetyl dopamine	390.676	34.986	294.186	13.841	96.490	1.328	P < 0.05
Other monoamines							
Tyramine	20.950	1.949	34.039	4.655	-13.089	0.615	P < 0.01
Serotonin	88.408	13.860	26.293	1.709	62.114	3.362	P < 0.001

* pmol/protein mg.

Mean values of F (n = 10) and G (n = 11) were used for calculations.

The levels of tyramine were significantly lower in foundresses than in newly emerged gynes (Table 1). The levels of serotonin were significantly higher in foundresses than in newly emerged gynes.

## Discussion

Temperate *Polistes* paper wasp species have different levels of caste differentiation with variations in caste determination [6,8,36]. Therefore, how the role of dopamine differs according to the evolution of eusociality and how dopamine is required in the flexible society of paper wasps requires further clarification. In these species, intermediate or undifferentiated characteristics in the brains between castes are expected. In the present study, we discovered caste differences in the levels of dopamine-related substances in the brains of four *Polistes* species and the storage of dopamine precursors in the brains of emerged gynes in three species. We also reported the levels of dopamine-related substances in newly emerged gynes and foundresses in a *Polistes* species to discuss the usage of dopamine in gynes.

**Fig 4:**
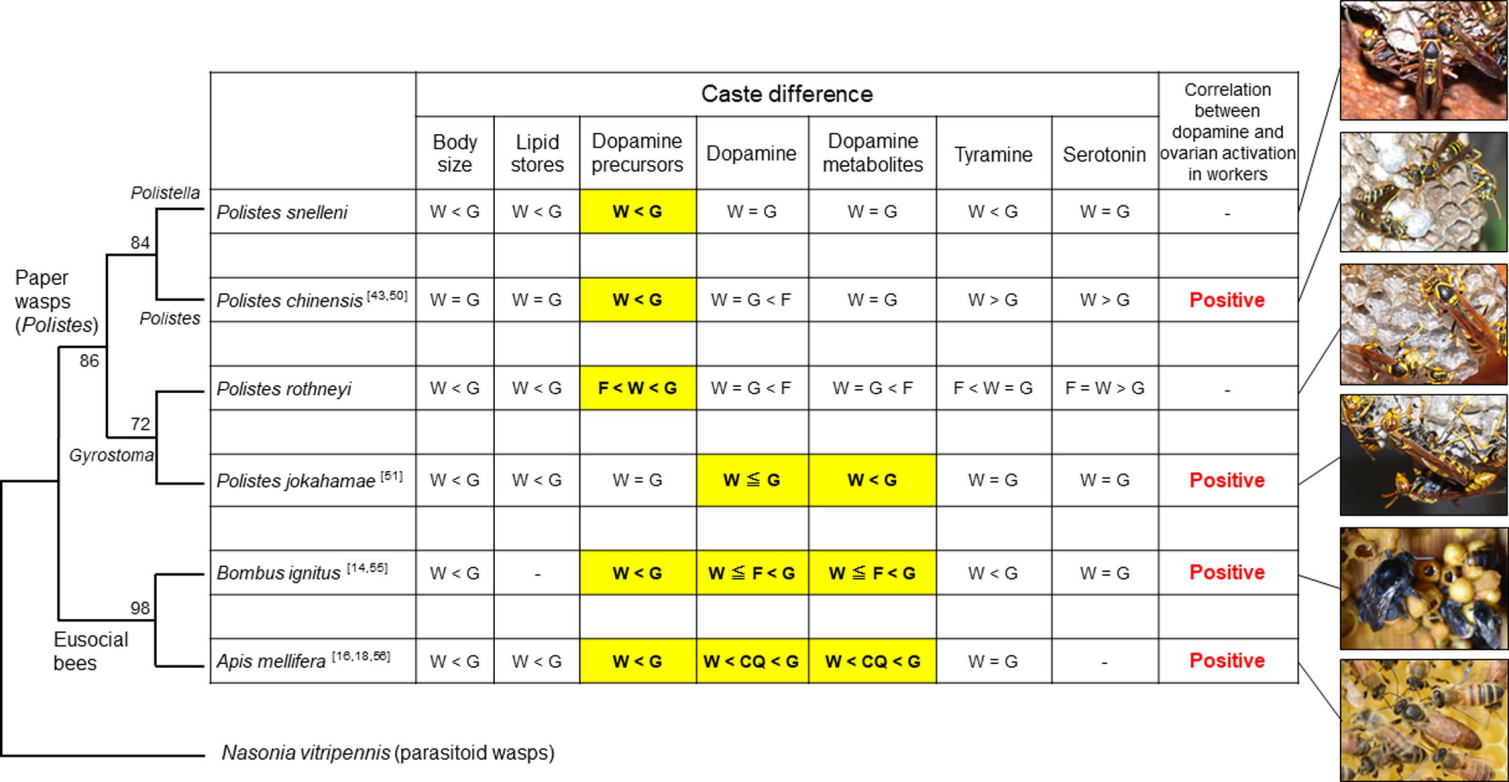
Comparison of caste differences in the body size, lipid stores and the levels of dopamine-related substances and other biogenic amines in the brains of four species of *Polistes* paper wasps and two species of eusocial bees. In the phylogenetic relationship, maximum likelihood trees were constructed using MEGA 11.0.10 software with 1,000-fold bootstrap re-sampling. The numbers at the nodes of the branches represent the levels of bootstrapping for each branch. *Nasonia vitripennis* 16S ribosomal RNA sequence was used as an outgroup. Dopamine precursors: tyrosine and L-DOPA, Dopamine metabolites: *N*-acetyldopamine and norepinephrine. CQ: colony queen, F: foundress, G: gyne, W: worker. Higher levels of dopamine-related substances in gynes than workers are highlighted as yellow. Reference papers are indicated as reference numbers beside the species name. Photos of the paper wasps and the eusocial bees were taken by M. Nishimura and K. Sasaki, respectively.

In *P*. *snelleni*, *P*. *chinensis*, and *P*. *rothneyi*, the levels of dopamine precursors, tyrosine and L-DOPA, in the brains were significantly larger in gynes than workers at their emergence, whereas the levels of dopamine and *N*-acetyldopamine did not differ between castes. These results suggested that gynes in these species stored large amounts of dopamine precursors in the brains and maintained the levels of dopamine and its metabolite at worker levels on emergence. These neuroendocrinal states largely differed from those in the honey bee and the bumble bee with the caste differences in dopamine levels in the brains (Fig 4). In *P*. *rothneyi*, the foundresses had significantly lower levels of tyrosine and L-DOPA, and higher levels of dopamine and *N*-acetyldopamine in comparison to newly emerged gynes, suggesting that dopamine might be used for behavior and physiology involved in colony founding. A similar higher level of dopamine in foundresses in comparison to normal workers has been reported in *P*. *chinensis* [50]. The dopamine levels in foundresses (approx. 80 pmol/brain in the study by Tsuchida et al. [50]) were about 5.3 times greater than in newly emerged gynes in the present study (approx. 15 pmol/brain in S1 Fig and S3 Table), indicating a similar rate of dopamine in *P*. *rothneyi* (5.519 times in Table 1). Thus, the storage of tyrosine and L-DOPA in the brains of emerged gynes in the three *Polistes* species might be an adaptive preparation for behavior and physiology during colony founding. However, we could find no information about the levels of dopamine-related substances in the brains of gynes at dispersion from the nest, after mating, and during hibernation. Since the neural development of adult brains with volume changes in various synaptic neuropils in *Polistes* wasps occurs early in adult life [37,38], the dopamine function in gynes’ behavior may change at different adult stages. Therefore, the investigation of the levels of dopamine-related substances and behavioral tests with pharmacological treatments using gynes at different adult stages are required.

In *P*. *jokahamae*, the levels of tyrosine and L-DOPA did not differ between castes, but the levels of *N*-acetyldopamine were significantly higher in gynes than workers at emergence. Although there were no significant differences in dopamine levels between castes, the levels tended to be higher in gynes than in workers. These results indicated that the storage levels of tyrosine and L-DOPA in the brains were similar between castes, but the conversion of dopamine and its metabolite was more active in gynes. This neuroendocrinal state is unique in comparison to the other three *Polistes* species, and is not due to phylogenetic constraints, because *P*. *rothneyi* with higher levels of dopamine precursors in gynes is a species that is closely related to *P*. *jokahamae* (Fig 4). Rather, this might be due to the high plasticity of ovarian activation depending on photoperiods in the adult females of *P*. *jokahamae* [31,51]. Adult females in *P*. *jokahamae* can activate ovaries under queenless long-day conditions, whereas they can store the lipid in the abdomen without ovarian activation under queenless short-day conditions [31]. Although the emerged gynes of *P*. *jokahamae* in the present study stored more lipids than workers, the gynes could change their physiological states to egg-laying females under queenless long-day conditions. On the contrary, the workers could change to gynes with more lipid stores for hibernation under short-day conditions. Both gynes and workers with activated ovaries under long-day conditions have higher dopamine and tyramine levels in the brains [51]. It has been reported that dopamine activated the ovaries in reproductive females in *P*. *chinensis* [52], honey bee *A*. *mellifera* [53], and several species of ants [21,22,54]. In the honey bee, dopamine receptor genes were expressed in ovarian tissue, and these expression levels differed between normal and reproductive workers [20], suggesting that dopamine directly acts on the ovarian tissue. This might be true in *P*. *jokahamae*. In *P*. *jokahamae*, gynes may keep levels of dopamine precursors similar to those of workers for plasticity to transform into egg-laying or hibernating females.

Dopamine roles in reproduction for queenless workers are shared between the *Polistes* paper wasps and the eusocial bees (Fig 4), but the dopamine function in gynes may differ between them. Foundresses in *P*. *rothneyi* had higher levels of dopamine and lower levels of dopamine precursors in brains than newly emerged gynes, suggesting that they may need dopamine for ovarian activation or egg-laying behavior as well as reproductive workers in *P*. *chinensis* [43,52] or other behaviors for colony founding. Thus, dopamine in the brains is thought to be related to ovarian activation in both workers and gynes in the *Polistes* species. However, it has been reported that foundresses in *B*. *ignitus* had lower levels of dopamine in the brain than in gynes one day after mating or in diapausing gynes [55]. Honey bee queens also had smaller levels of dopamine in egg-laying mated queens than in virgin gynes [18], although the levels of dopamine in both queens were still higher than those in workers. Thus, dopamine levels in the brains are associated with ovarian activation in workers but not in gynes of the bumble bee and the honey bee. The share of dopamine function between castes in *Polistes* and the different dopamine function between castes in eusocial bees may be based on phylogenetical differences and associated with degrees of caste differentiation or plasticity of caste determination in adults: the caste in *Polistes* is less differentiated and more plastic than that in the bumble bee and the honey bee. More information on caste differences in the levels of dopamine-related substances in the brains of other paper wasps and hornets with advanced eusociality is required for the examination of phylogenetical constraints and convergent neuroendocrinal states on each caste.

In *P*. *snelleni* and *P*. *chinensis*, differences in tyramine levels in the brains were found between workers and gynes. However, gynes had higher tyramine levels in *P*. *snelleni*, whereas workers had higher tyramine levels in *P*. *chinensis*. Because tyramine is synthesized from tyrosine, the higher levels of tyrosine in gynes might influence tyramine levels in gynes of *P*. *snelleni*. However, we could not explain the higher tyramine levels in workers of *P*. *chinensis*. Serotonin is synthesized in a pathway that is independent of the synthetic pathways of dopamine and tyramine [11,14]. Higher levels of serotonin in workers were observed in *P*. *chinensis* and *P*. *rothneyi*. In *P*. *chinensis*, serotonin levels were positively correlated with ovarian diameters in workers under queenright conditions [43]. The higher serotonin levels in workers at emergence might influence subsequent ovarian development or reproductive hierarchy among workers. The differences in the levels of tyramine and serotonin might be species-specific and the physiological significance should be investigated in the future.

Among the four *Polistes* species, the head widths and the lipid stores were different between castes in *P*. *snelleni*, *P*. *rothneyi*, and *P*. *jokahamae*, but not in *P*. *chinensis*. The former meets the criteria on time of collection and lipid stores to distinguish gynes from workers, suggested by Toth *et al*. [46], but the latter is only based on the time of collection. In *P*. *chinensis*, workers have developed ovaries under queenright conditions [41–43], which differs from the other three species. Since the workers with developed ovaries do not mate with males, workers contribute only a part of male production [44]. Thus, eusociality in *P*. *chinensis* is more primitive than in the other three species, which may be associated with the lack of caste differences in the head widths and lipid stores. However, the levels of dopamine precursors differed between castes in *P*. *chinensis*. Therefore, the caste differences in neuroendocrinal states at emergence in *Polistes* did not always match body sizes and nutritional reserves. Because the gynes and workers were collected from different developmental stages of the colony with different external environmental conditions in this study, this factor might influence neuroendocrinal states, even though the body sizes and nutritional reserves were similar between castes.

## Conclusion

This study investigated caste differences in the neuroendocrinal states of brains at emergence in four *Polistes* species and during colony founding in a *Polistes* species. We revealed that the levels of dopamine precursors in the brains were higher in gynes than workers at emergence in three *Polistes* species, whereas those of dopamine and its metabolite did not differ between castes in these species. Dopamine precursors were stored in the brains without dopamine biosynthesis at emergence, and then were converted into dopamine in foundresses during colony founding. The storage of dopamine precursors in the brains of emerged gynes in the three *Polistes* species might be an adaptive preparation for behavior and physiology during colony founding. These characteristics in the paper wasps largely differed from those in eusocial bees with high levels of dopamine in the emerged gynes. This may be based on phylogenetical differences and associated with degrees of caste differentiation or plasticity of caste determination in adults.

## Supporting information

S1 Fig Unnormalized levels of dopamine-related substances and other monoamines in the brains of newly emerged workers and gynes of four species of *Polistes* paper wasps. (PDF)

S1 Table Data of head width and lipid stores in four *Polistes* species. (PDF)

S2 Table Data of monoamine levels in the brain of *Polistes snelleni*. (PDF)

S3 Table Data of monoamine levels in the brain of *Polistes chinensis*. (PDF)

S4 Table Data of monoamine levels in the brain of *Polistes rothneyi*. (PDF)

S5 Table Data of monoamine levels in the brain of *Polistes jokahamae*. (PDF)

S6 Table Tests of normality and equal variance in each group. (PDF)
