# Supplementary material for: Caste-specific storage of dopamine-related substances in the brains of four Polistes paper wasp species
Source: PLoS One. 2023 Jan 26;18(1):e0280881. doi: 10.1371/journal.pone.0280881 (PMC9879392; doi:10.1371/journal.pone.0280881)
Supplement: S1 Fig — (PDF) [file pone.0280881.s001.pdf]

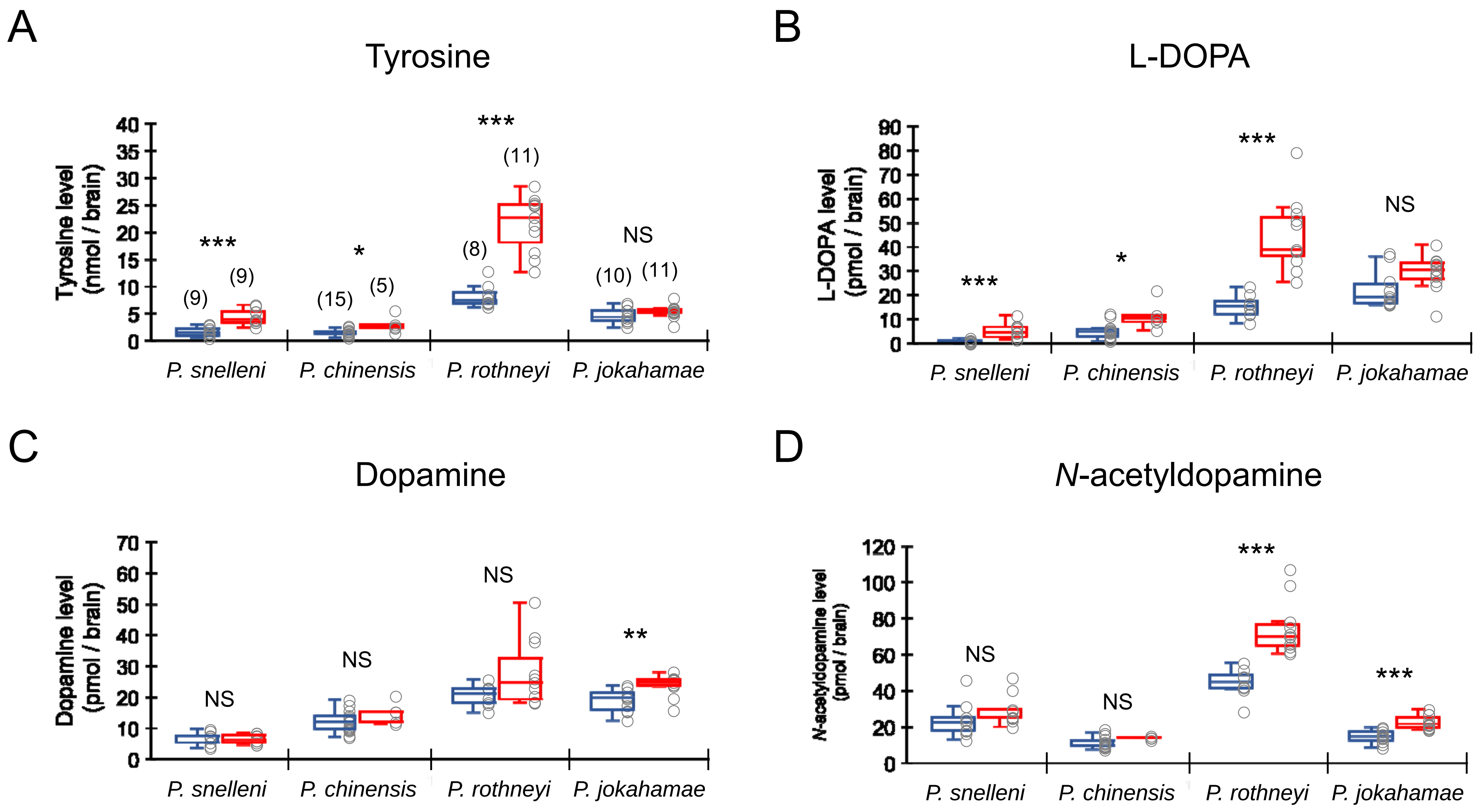
A
Tyrosine
Tyrosine level (nmol / brain)
*** * *** NS
(9) (9) (15) (5) (8) (11) (10) (11)
B
L-DOPA
L-DOPA level (pmol / brain)
*** * *** NS
C
Dopamine
Dopamine level (pmol / brain)
NS NS NS **
D
N-acetyldopamine
N-acetyldopamine level (pmol / brain)
NS NS *** ***
P. snelleni
P. chinensis
P. rothneyi
P. jokahamae

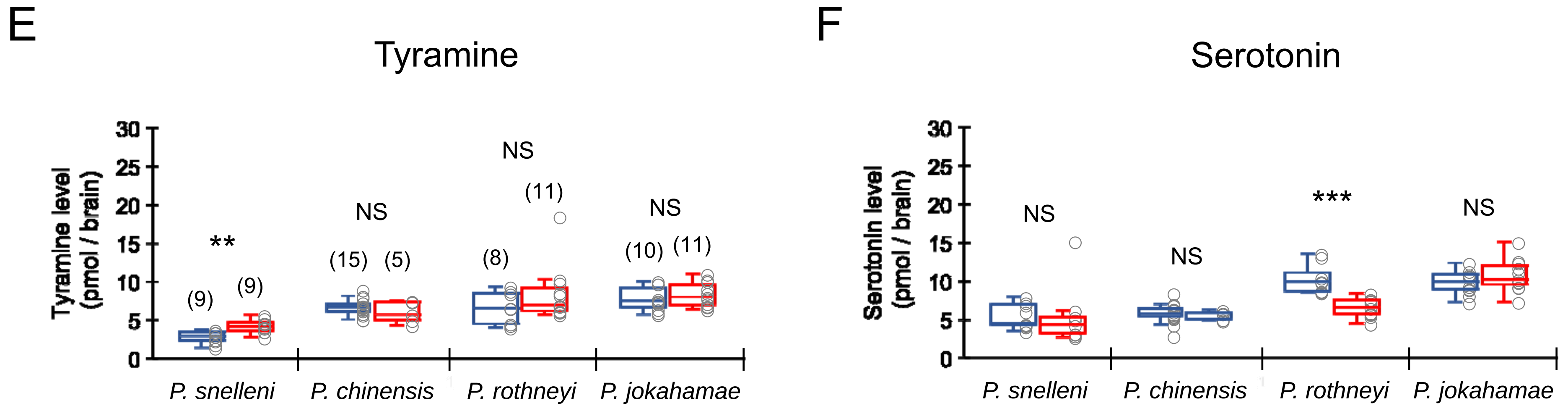

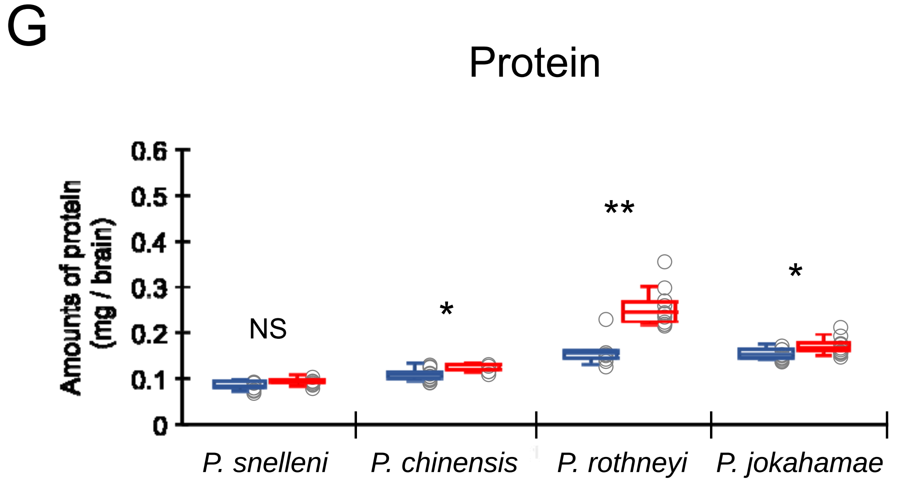

S1 Fig. Unnormalized levels of dopamine-related substances and other monoamines in the brains of newly emerged workers and gynes of four species of *Polistes* paper wasps. (A) Tyrosine levels, (B) L-DOPA levels, (C) Dopamine levels, (D) *N*-acetyldopamine levels, (E) Tyramine levels, (F) Serotonin levels and (G) Amounts of protein in the brain. Blue and red box plots indicate workers and gynes, respectively. Numbers in parentheses in the graph indicate the numbers of samples examined. The numbers of samples are the same for six substances and protein. Asterisks in the graph indicate significant differences between workers and gynes (*: P < 0.05, **: P < 0.01, ***: P < 0.001, NS: not significant, P > 0.05).
