# Supplementary material for: Caste-specific storage of dopamine-related substances in the brains of four Polistes paper wasp species
Source: PLoS One. 2023 Jan 26;18(1):e0280881. doi: 10.1371/journal.pone.0280881 (PMC9879392; doi:10.1371/journal.pone.0280881)
Supplement: S1 Table — (PDF) [file pone.0280881.s002.pdf]

S1 Table. Data of head width and lipid stores in four *Polistes* species

Species	Colony ID	Caste	Head width	Lipid stores
Polistes snelleni	S20004	Worker	3.12	0.7
Polistes snelleni	S20004	Worker	3.19	0.6
Polistes snelleni	S20004	Worker	3.19	1.1
Polistes snelleni	S20004	Worker	3.21	1.4
Polistes snelleni	S20004	Worker	3.18	0.8
Polistes snelleni	S20005	Worker	3.17	0.8
Polistes snelleni	S20005	Worker	3.08	0.6
Polistes snelleni	S20006	Worker	3.09	1.6
Polistes snelleni	S20006	Worker	3.11	2.1
Polistes snelleni	S20006	Worker	3.19	2.2
Polistes snelleni	S20005	Worker	2.95	0.6
Polistes snelleni	S20005	Worker	3.24	1.5
Polistes snelleni	P19012	Gyne	3.21	0.6
Polistes snelleni	P19012	Gyne	3.18	0.7
Polistes snelleni	P19012	Gyne	3.16	0.8
Polistes snelleni	P19012	Gyne	3.2	0.9
Polistes snelleni	P19012	Gyne	3.24	1
Polistes snelleni	P19016	Gyne	3.26	2.6
Polistes snelleni	P19016	Gyne	3.33	2.1
Polistes snelleni	P19016	Gyne	3.3	3.1
Polistes snelleni	P19016	Gyne	3.25	2.6
Polistes snelleni	P19012	Gyne	3.19	0.9
Polistes snelleni	P19016	Gyne	3.28	2.8
Polistes snelleni	P19016	Gyne	3.25	2.1
Polistes snelleni	P19016	Gyne	3.26	2.3
Polistes snelleni	P19016	Gyne	3.22	2.7
Polistes snelleni	P19016	Gyne	3.26	3.8
Polistes snelleni	P19016	Gyne	3.27	2.5
Polistes snelleni	P19016	Gyne	3.22	2.2
Polistes snelleni	P19016	Gyne	3.28	2.5
Polistes snelleni	P19016	Gyne	3.31	2.7
Polistes snelleni	P19016	Gyne	3.25	2.5
Polistes snelleni	P19016	Gyne	3.33	2.5
Polistes snelleni	P19016	Gyne	3.27	2.1
Polistes snelleni	P19016	Gyne	3.3	2.8
Polistes snelleni	P19016	Gyne	3.15	1.4
Polistes snelleni	P19016	Gyne	3.24	3.2
Polistes snelleni	P19016	Gyne	3.26	3.4
Polistes snelleni	P19016	Gyne	3.26	2
Polistes snelleni	P19016	Gyne	3.16	1.2
Polistes snelleni	P19016	Gyne	3.3	2.4
Polistes snelleni	P19016	Gyne	3.27	2.4
Polistes snelleni	P19016	Gyne	3.32	2.6
Polistes snelleni	P19016	Gyne	3.33	2.8
Polistes snelleni	P19016	Gyne	3.33	2.3
Polistes snelleni	P19016	Gyne	3.35	3.5
Polistes snelleni	P19016	Gyne	3.32	2.7
Polistes snelleni	P19016	Gyne	3.29	1
Polistes snelleni	P19016	Gyne	3.28	4
Polistes snelleni	P19016	Gyne	3.2	3.7
Polistes snelleni	P19016	Gyne	3.32	3
Polistes snelleni	P19016	Gyne	3.2	3.6
Polistes snelleni	P19016	Gyne	3.18	4
Polistes snelleni	P19016	Gyne	3.25	3.2
Polistes snelleni	P19016	Gyne	3.2	4.1
Polistes snelleni	P19016	Gyne	3.23	4.3
Polistes snelleni	P19016	Gyne	3.18	3.2
Polistes snelleni	P19016	Gyne	3.16	3.5
Polistes snelleni	P19016	Gyne	3.21	3.6
Polistes snelleni	P19016	Gyne	3.21	3.6
Polistes snelleni	P19016	Gyne	3.25	3
Polistes snelleni	P19016	Gyne	3.18	2.8
Polistes snelleni	P19016	Gyne	3.24	3.7
Polistes snelleni	S20004	Gyne	3.22	1.3
Polistes snelleni	S20004	Gyne	3.18	1.2
Polistes snelleni	S20004	Gyne	3.22	1.4
Polistes snelleni	S20006	Gyne	3.16	1.5
Polistes snelleni	S20006	Gyne	3.16	1.5
Polistes snelleni	S20009	Gyne	2.95	0.4
Polistes snelleni	S20007	Gyne	3.37	2.4
Polistes snelleni	S20007	Gyne	3.39	1.6
Polistes snelleni	S20008	Gyne	3.39	4.3
Polistes snelleni	S20008	Gyne	3.23	2.8
Polistes snelleni	S20008	Gyne	3.25	3
Polistes snelleni	S20008	Gyne	3.35	3.4
Polistes snelleni	S20007	Gyne	3.39	2.1
Polistes snelleni	S20008	Gyne	3.41	2.3
Polistes snelleni	S20008	Gyne	3.4	2.5
Polistes snelleni	S20007	Gyne	3.28	1.1
Polistes snelleni	S20008	Gyne	3.39	2.8
Polistes snelleni	S20008	Gyne	3.39	2.3
Polistes snelleni	S20008	Gyne	3.41	2.5
Polistes snelleni	S20008	Gyne	3.35	2.3
Polistes snelleni	S20009	Gyne	3.2	2.3
Polistes snelleni	S20009	Gyne	3.32	1.8
Polistes snelleni	S20007	Gyne	3.31	2
Polistes snelleni	S20007	Gyne	3.32	1.5
Polistes snelleni	S20007	Gyne	3.28	1.9
Polistes snelleni	S20008	Gyne	3.25	2.1
Polistes snelleni	S20008	Gyne	3.38	2.9
Polistes snelleni	S20008	Gyne	3.31	2.8
Polistes snelleni	S20008	Gyne	3.24	1.8
Polistes snelleni	S20008	Gyne	3.3	2.2
Polistes snelleni	S20009	Gyne	3.32	2.6
Polistes snelleni	S20007	Gyne	3.35	0.8
Polistes snelleni	S20007	Gyne	3.37	1
Polistes snelleni	S20008	Gyne	3.31	1.2
Polistes snelleni	S20008	Gyne	3.27	0.4
Polistes snelleni	S20008	Gyne	3.25	0.8
Polistes snelleni	S20008	Gyne	3.31	0.8
Polistes snelleni	S20009	Gyne	3.39	3
Polistes snelleni	S20009	Gyne	3.3	1.3
Polistes snelleni	S20009	Gyne	3.22	3
Polistes snelleni	S20009	Gyne	3.25	1.1
Polistes snelleni	S20007	Gyne	3.33	2.1
Polistes snelleni	S20007	Gyne	3.27	2
Polistes snelleni	S20007	Gyne	3.26	1.5
Polistes snelleni	S20007	Gyne	3.21	0.8
Polistes snelleni	S20008	Gyne	3.35	1.9
Polistes snelleni	S20008	Gyne	3.35	2.2
Polistes snelleni	S20009	Gyne	3.25	1.9
Polistes snelleni	S20009	Gyne	3.24	1.9
Polistes snelleni	S20007	Gyne	3.25	1.2
Polistes snelleni	S20007	Gyne	3.27	1.7
Polistes snelleni	S20007	Gyne	3.23	1.4
Polistes snelleni	S20007	Gyne	3.28	0.9
Polistes snelleni	S20008	Gyne	3.26	3.8
Polistes snelleni	S20008	Gyne	3.3	3.8
Polistes snelleni	S20009	Gyne	3.34	1.3
Polistes snelleni	S20009	Gyne	3.4	2.4
Polistes snelleni	S20007	Gyne	3.26	1.8
Polistes snelleni	S20007	Gyne	3.13	1.3
Polistes snelleni	S20007	Gyne	3.17	0.2
Polistes snelleni	S20007	Gyne	3.19	1.3
Polistes snelleni	S20007	Gyne	3.29	1.9
Polistes snelleni	S20007	Gyne	3.2	1.8
Polistes snelleni	S20008	Gyne	3.19	1.7
Polistes snelleni	S20009	Gyne	3.35	1.8
Polistes snelleni	S20009	Gyne	3.24	0.9
Polistes snelleni	S20009	Gyne	3.23	0.5
Polistes snelleni	S20009	Gyne	3.2	1.6
Polistes snelleni	S20009	Gyne	3.37	1.8

Species	Colony ID	Caste	Head width	Lipid stores
Polistes chinensis	P19010	Worker	3.27	0.9
Polistes chinensis	P19009	Worker	3.24	1.4
Polistes chinensis	P19011	Worker	3.11	1.5
Polistes chinensis	P19011	Worker	2.97	0.6
Polistes chinensis	P19011	Worker	3.15	0.7
Polistes chinensis	P19011	Worker	3.22	1
Polistes chinensis	P19010	Worker	3.29	0.4
Polistes chinensis	P19011	Worker	3.3	0.3
Polistes chinensis	P19011	Worker	3.33	0.6
Polistes chinensis	P19010	Worker	3.25	0.9
Polistes chinensis	P19010	Worker	3.42	0.4
Polistes chinensis	P19010	Worker	3.37	0.7
Polistes chinensis	P19010	Worker	3.27	0.6
Polistes chinensis	P19010	Worker	3.12	0.2
Polistes chinensis	P19010	Worker	3.13	0.3
Polistes chinensis	P19010	Worker	3.16	0.9
Polistes chinensis	P19010	Worker	3.15	0.5
Polistes chinensis	P19010	Worker	3.19	0.4
Polistes chinensis	P19010	Worker	3.14	0.5
Polistes chinensis	P19014	Worker	3.51	0.7
Polistes chinensis	P19014	Worker	3.49	1.2
Polistes chinensis	P19014	Worker	3.61	1.2
Polistes chinensis	P19014	Worker	3.55	1.4
Polistes chinensis	P19014	Worker	3.56	1.4
Polistes chinensis	P19014	Worker	3.56	1.2
Polistes chinensis	P20013	Worker	3.24	0.6
Polistes chinensis	P20015	Worker	3.36	0.3
Polistes chinensis	P20015	Worker	3.43	0.5
Polistes chinensis	P20015	Worker	3.48	0.1
Polistes chinensis	P20014	Worker	3.04	0.1
Polistes chinensis	P20014	Worker	3.02	0.2
Polistes chinensis	P20015	Worker	3.44	0.2
Polistes chinensis	P20015	Worker	3.49	0.7
Polistes chinensis	P20015	Worker	3.51	0.8
Polistes chinensis	P20016	Worker	3.1	0.7
Polistes chinensis	P20014	Worker	2.95	0.1
Polistes chinensis	P20015	Worker	3.59	0.8
Polistes chinensis	P20016	Worker	3.14	0.4
Polistes chinensis	P20015	Worker	3.55	1.7
Polistes chinensis	P20015	Worker	3.67	2
Polistes chinensis	P20016	Worker	3.27	0.7
Polistes chinensis	P20016	Worker	3.33	0.2
Polistes chinensis	P20016	Worker	3.19	0.5
Polistes chinensis	P20016	Worker	3.24	0.3
Polistes chinensis	P20015	Worker	3.65	2.1
Polistes chinensis	P20015	Worker	3.59	2.1
Polistes chinensis	P20015	Worker	3.61	2.5
Polistes chinensis	P20015	Worker	3.61	2
Polistes chinensis	P20015	Worker	3.51	2.4
Polistes chinensis	P20015	Worker	3.56	1.8
Polistes chinensis	P20015	Worker	3.55	0.8
Polistes chinensis	P20015	Worker	3.61	1.2
Polistes chinensis	P20015	Worker	3.6	0.8
Polistes chinensis	P20015	Worker	3.62	0.6
Polistes chinensis	P20015	Worker	3.6	1.2
Polistes chinensis	P20015	Worker	3.6	1.1
Polistes chinensis	P20015	Worker	3.63	3.3
Polistes chinensis	P20015	Worker	3.6	2.8
Polistes chinensis	P20015	Worker	3.5	3.1
Polistes chinensis	P20015	Worker	3.56	3.6
Polistes chinensis	P20016	Worker	3.48	0.8
Polistes chinensis	P20017	Worker	3.3	1.4
Polistes chinensis	P20017	Worker	3.35	1.1
Polistes chinensis	P20017	Worker	3.28	1
Polistes chinensis	P20017	Worker	3.4	0.8
Polistes chinensis	P20017	Worker	3.44	0.9
Polistes chinensis	P20017	Worker	3.49	1
Polistes chinensis	P20017	Worker	3.55	1
Polistes chinensis	P20018	Worker	3.47	0.9
Polistes chinensis	P20018	Worker	3.56	1.4
Polistes chinensis	P20018	Worker	3.59	1.7
Polistes chinensis	P20017	Worker	3.47	1.4
Polistes chinensis	P20017	Worker	3.41	1.6
Polistes chinensis	P20017	Worker	3.53	2.5
Polistes chinensis	P20017	Worker	3.54	2
Polistes chinensis	P20018	Worker	3.52	1.3
Polistes chinensis	P20018	Worker	3.53	2
Polistes chinensis	P20018	Worker	3.59	1.1
Polistes chinensis	P20018	Worker	3.59	1.9
Polistes chinensis	P20018	Worker	3.57	2.9
Polistes chinensis	P20018	Worker	3.6	3
Polistes chinensis	P20018	Worker	3.56	2.6
Polistes chinensis	P20018	Worker	3.59	1.3
Polistes chinensis	P19013	Gyne	3.35	1.7
Polistes chinensis	P19013	Gyne	3.43	1.6
Polistes chinensis	P19013	Gyne	3.41	1.7
Polistes chinensis	P19013	Gyne	3.44	1.9
Polistes chinensis	P19013	Gyne	3.4	1.5
Polistes chinensis	P19013	Gyne	3.38	1.3
Polistes chinensis	P19013	Gyne	3.42	1.7
Polistes chinensis	P19013	Gyne	3.4	1.1
Polistes chinensis	P19013	Gyne	3.43	1.2
Polistes chinensis	P19013	Gyne	3.42	0.8
Polistes chinensis	P19013	Gyne	3.38	1.6
Polistes chinensis	P19013	Gyne	3.46	1.4
Polistes chinensis	P19013	Gyne	3.46	4.8
Polistes chinensis	P19013	Gyne	3.35	0.9
Polistes chinensis	P19013	Gyne	3.24	0.7
Polistes chinensis	P19013	Gyne	3.3	0.9
Polistes chinensis	P19013	Gyne	3.24	1.1
Polistes chinensis	P19013	Gyne	3.29	1.2
Polistes chinensis	P19013	Gyne	3.37	1
Polistes chinensis	P19013	Gyne	3.38	0.8
Polistes chinensis	P19014	Gyne	3.58	1
Polistes chinensis	P19013	Gyne	3.43	0.9
Polistes chinensis	P19013	Gyne	3.56	0.8
Polistes chinensis	P19013	Gyne	3.47	0.8
Polistes chinensis	P19014	Gyne	3.51	0.7
Polistes chinensis	P19013	Gyne	3.34	0.8
Polistes chinensis	P19013	Gyne	3.46	1

Species	Colony ID	Caste	Head width	Lipid stores
Polistes rothneyi	R20001	Worker	4.6	2
Polistes rothneyi	R20001	Worker	4.66	3.6
Polistes rothneyi	R20001	Worker	4.69	3.9
Polistes rothneyi	R20001	Worker	4.73	2.3
Polistes rothneyi	R20001	Worker	4.54	3.1
Polistes rothneyi	R20001	Worker	4.48	1.5
Polistes rothneyi	R20001	Worker	4.25	2.4
Polistes rothneyi	R20002	Worker	4.28	0.9
Polistes rothneyi	R20002	Worker	4.62	5
Polistes rothneyi	R20005	Gyne	4.92	10.1
Polistes rothneyi	R20005	Gyne	4.82	12.3
Polistes rothneyi	R20005	Gyne	4.94	10.8
Polistes rothneyi	R20005	Gyne	4.84	10.2
Polistes rothneyi	R20005	Gyne	4.8	8.9
Polistes rothneyi	R20005	Gyne	4.87	8.1
Polistes rothneyi	R20005	Gyne	4.83	8.5
Polistes rothneyi	R20006	Gyne	4.87	4.9
Polistes rothneyi	R20007	Gyne	4.9	9.5
Polistes rothneyi	R20008	Gyne	4.78	3.9
Polistes rothneyi	R20008	Gyne	4.78	2.9
Polistes rothneyi	R20008	Gyne	4.83	3.6
Polistes rothneyi	R20006	Gyne	4.83	5.9
Polistes rothneyi	R20006	Gyne	4.88	6.1
Polistes rothneyi	R20006	Gyne	4.88	7.4
Polistes rothneyi	R20007	Gyne	4.95	12
Polistes rothneyi	R20007	Gyne	4.89	11
Polistes rothneyi	R20007	Gyne	4.98	10.7
Polistes rothneyi	R20007	Gyne	4.9	9.8
Polistes rothneyi	R20006	Gyne	4.92	6.7
Polistes rothneyi	R20006	Gyne	4.86	5.3
Polistes rothneyi	R20006	Gyne	4.92	7.1
Polistes rothneyi	R20006	Gyne	5	9.3
Polistes rothneyi	R20006	Gyne	4.93	5.6
Polistes rothneyi	R20007	Gyne	4.75	7.1
Polistes rothneyi	R20006	Gyne	4.95	8.9
Polistes rothneyi	R20006	Gyne	4.75	7.2
Polistes rothneyi	R20006	Gyne	4.9	7.8
Polistes rothneyi	R20006	Gyne	4.92	6.7
Polistes rothneyi	R20006	Gyne	4.94	8.3
Polistes rothneyi	R20006	Gyne	4.73	3.4
Polistes rothneyi	R20006	Gyne	4.87	4.9
Polistes rothneyi	R20006	Gyne	4.91	6.7
Polistes rothneyi	R20007	Gyne	4.83	5.5
Polistes rothneyi	R20007	Gyne	4.99	6.3
Polistes rothneyi	R20006	Gyne	4.7	8.4

Species	Colony ID	Caste	Head width	Lipid stores
Polistes jokahamae	J20015	Worker	4.18	1.3
Polistes jokahamae	J20015	Worker	4.33	1.1
Polistes jokahamae	J20015	Worker	4.23	0.5
Polistes jokahamae	J20015	Worker	4.22	1.6
Polistes jokahamae	J20015	Worker	4.47	3.6
Polistes jokahamae	J20014	Worker	4.45	1.3
Polistes jokahamae	J20016	Worker	4.70	5.2
Polistes jokahamae	J20016	Worker	4.68	11.4
Polistes jokahamae	J20022	Worker	4.23	1.6
Polistes jokahamae	J20019	Worker	4.62	10.8
Polistes jokahamae	J20023	Worker	4.79	4.1
Polistes jokahamae	J20029	Worker	4.72	8.1
Polistes jokahamae	J20029	Worker	4.64	5.3
Polistes jokahamae	J20015	Worker	4.40	4.5
Polistes jokahamae	J20016	Worker	4.71	8.7
Polistes jokahamae	J20018	Worker	4.76	15.4
Polistes jokahamae	J20020	Worker	4.39	5.7
Polistes jokahamae	J20023	Worker	4.75	9.4
Polistes jokahamae	J20026	Worker	4.74	5.6
Polistes jokahamae	J20030	Worker	4.68	7.2
Polistes jokahamae	J20015	Worker	4.48	3.1
Polistes jokahamae	J20015	Worker	4.31	3.8
Polistes jokahamae	J20018	Worker	4.71	14.5
Polistes jokahamae	J20023	Worker	4.88	8.1
Polistes jokahamae	J20026	Worker	4.70	7
Polistes jokahamae	J20016	Worker	4.83	10
Polistes jokahamae	J20016	Worker	4.69	7.5
Polistes jokahamae	J20016	Worker	4.67	7.4
Polistes jokahamae	J20016	Worker	4.79	8
Polistes jokahamae	J20018	Worker	4.73	13.1
Polistes jokahamae	J20024	Worker	4.50	3.9
Polistes jokahamae	J20020	Worker	4.44	6.4
Polistes jokahamae	J20014	Worker	4.87	2.2
Polistes jokahamae	J20014	Worker	4.81	0.8
Polistes jokahamae	J20015	Worker	4.44	3.6
Polistes jokahamae	J20026	Worker	4.75	9.3
Polistes jokahamae	J20026	Worker	4.84	9
Polistes jokahamae	J20027	Worker	4.78	9.3
Polistes jokahamae	J20026	Worker	4.52	7.7
Polistes jokahamae	J20028	Worker	4.52	1.1
Polistes jokahamae	J20026	Worker	4.71	8.5
Polistes jokahamae	J20021	Worker	4.27	4.7
Polistes jokahamae	J20020	Worker	4.01	4.2
Polistes jokahamae	J20016	Gyne	4.90	8.3
Polistes jokahamae	J20016	Gyne	4.90	8.7
Polistes jokahamae	J20016	Gyne	4.84	9.6
Polistes jokahamae	J20016	Gyne	4.71	7.9
Polistes jokahamae	J20016	Gyne	4.88	8.5
Polistes jokahamae	J20016	Gyne	4.88	13.6
Polistes jokahamae	J20016	Gyne	4.75	5
Polistes jokahamae	J20016	Gyne	4.77	9.4
Polistes jokahamae	J20016	Gyne	4.74	10.8
Polistes jokahamae	J20016	Gyne	4.65	8.8
Polistes jokahamae	J20016	Gyne	4.83	13.3
Polistes jokahamae	J20016	Gyne	4.50	6.9
Polistes jokahamae	J20024	Gyne	4.48	6
Polistes jokahamae	J20016	Gyne	4.65	7.9
Polistes jokahamae	J20016	Gyne	4.77	12.3
Polistes jokahamae	J20023	Gyne	4.66	4.6
Polistes jokahamae	J20016	Gyne	4.61	9.6
Polistes jokahamae	J20019	Gyne	4.72	12.9
