# Supplementary material for: Caste-specific storage of dopamine-related substances in the brains of four Polistes paper wasp species
Source: PLoS One. 2023 Jan 26;18(1):e0280881. doi: 10.1371/journal.pone.0280881 (PMC9879392; doi:10.1371/journal.pone.0280881)
Supplement: S2 Table — (PDF) [file pone.0280881.s003.pdf]

S2 Table. Data of monoamine levels in the brain of *Polistes snelleni* .

		pmol/brain								nmol/protein mg	pmol/protein mg				
Worker	Colony	Tyr	DOPA	DA	NADA	TA	5HT	Protein mg		Tyr	DOPA	DA	NADA	TA	5HT
1	S20005	1014.469	0.027261	9.731472	31.57955	2.995671	4.389839	0.077726073		13.05185	0.350733	125.2022	406.2929	38.54139	56.47834
2	S20004	1523.508	0.28397	4.292906	18.59335	2.389298	4.331993	0.096054448		15.86088	2.956345	44.69242	193.5709	24.87441	45.09935
3	S20002	969.3658	0.095414	6.957863	12.93639	3.497431	4.488413	0.07311821		13.25752	1.304927	95.1591	176.9243	47.83255	61.3857
4	S20005	2045.936	0.8769	5.439664	24.14463	1.470102	4.159604	0.085057495		24.05356	10.30949	63.95279	283.8625	17.28362	48.90344
5	S20004	481.069	0.002272	3.696601	18.17591	1.896307	3.524352	0.084713149		5.678799	0.026817	43.63668	214.5583	22.38503	41.60336
6	S20003	1398.411	0.387019	7.592483	16.76823	2.544051	7.28597	0.080148844		17.44768	4.82875	94.72979	209.2136	31.74158	90.90549
7	S20003	2326.974	1.121387	9.138659	25.48581	3.513548	6.080848	0.096286355		24.16723	11.64637	94.91126	264.6876	36.49061	63.15379
8	S20001	3070.98	2.331788	7.526862	46.1654	3.524194	7.96192	0.094943018		32.3455	24.55986	79.27767	486.2433	37.11904	83.85998
9	S20001	2861.928	1.424229	7.697946	22.73041	3.816387	6.974961	0.097592606		29.32525	14.59361	78.87838	232.9111	39.10529	71.47018
mean		1743.627	0.727804	6.897162	24.06441	2.849665	5.466433	0.087293355		19.46536	7.841879	80.04892	274.2516	32.81928	62.53996

		pmol/brain								nmol/protein mg	pmol/protein mg				
Gyne	Colony	Tyr	DOPA	DA	NADA	TA	5HT	Protein mg		Tyr	DOPA	DA	NADA	TA	5HT
1	S20021	3740.587	2.709074	5.410397	23.76099	2.782365	2.994438	0.083651609		44.71626	32.3852	64.67775	284.047	33.26134	35.79654
2	S20021	6659.339	6.911831	7.745497	25.14456	4.177218	4.366599	0.094927176		70.15208	72.81194	81.5941	264.8826	44.00444	45.99947
3	S20021	3414.345	2.598894	4.731415	29.17159	4.714868	2.764295	0.09103113		37.50744	28.54951	51.97579	320.4573	51.79402	30.36648
4	S20021	5427.92	7.308254	8.561788	40.5553	4.04334	6.230018	0.107791721		50.35563	67.79977	79.429	376.2376	37.51068	57.79681
5	S20022	6447.745	11.68253	7.555875	25.72364	3.590421	5.408387	0.091798619		70.23793	127.2626	82.30924	280.2182	39.11193	58.91578
6	S20021	2456.583	1.565491	4.748925	20.10228	3.568018	3.264792	0.0981359		25.03246	15.95227	48.39132	204.8413	36.35792	33.26807
7	S20021	3412.562	4.564885	8.482444	47.40908	4.597772	5.060157	0.091523241		37.28629	49.87679	92.68076	518.0005	50.23612	55.28822
8	S20021	5382.603	6.370328	6.052844	25.49517	5.310838	3.207079	0.099654215		54.01279	63.92432	60.73847	255.8363	53.29266	32.18207
9	S20021	3943.521	4.411046	6.104651	29.9561	5.687681	15.20319	0.095083188		41.47443	46.39144	64.20326	315.0515	59.81795	159.8936
mean		4542.801	5.346926	6.599315	29.70208	4.274725	5.388773	0.094844089		47.86392	56.10598	69.55552	313.2858	45.04301	56.61189
