# Supplementary material for: Caste-specific storage of dopamine-related substances in the brains of four Polistes paper wasp species
Source: PLoS One. 2023 Jan 26;18(1):e0280881. doi: 10.1371/journal.pone.0280881 (PMC9879392; doi:10.1371/journal.pone.0280881)
Supplement: S3 Table — (PDF) [file pone.0280881.s004.pdf]

S3 Table. Data of monoamine levels in the brain of *Polistes chinensis* .

		pmol/brain								nmol/protein mg	pmol/protein mg				
Worker	Colony	Tyr	DOPA	DA	NADA	TA	5HT	Protein mg		Tyr	DOPA	DA	NADA	TA	5HT
1	C21001	1396.7	4.387518	7.161561	11.88825	5.14123	2.892403	0.104693359		13.34087	41.90827	68.40512	113.553	49.10751	27.62737
2	C21001	1415.419	1.681908	9.287423	7.734352	7.338903	5.756391	0.116384301		12.1616	14.45133	79.79962	66.45528	63.0575	49.46021
3	C21002	1697.134	5.023306	9.839621	15.77863	6.918625	6.064129	0.10999858		15.42869	45.66701	89.45226	143.444	62.8974	55.12915
4	C21001	1732.641	6.410316	10.20151	8.838418	8.971251	5.528264	0.114889332		15.08096	55.79557	88.7942	76.92984	78.08602	48.11817
5	C21003	581.9645	2.445111	12.0818	9.929006	6.680443	5.490824	0.096509363		6.030135	25.33548	125.1879	102.8813	69.22067	56.89421
6	C21002	1215.703	4.897952	9.637999	10.06114	8.192785	5.360318	0.096513834		12.59615	50.74871	99.86132	104.2456	84.88716	55.53937
7	C21003	1798.998	5.230726	12.30895	9.764407	6.835814	7.08178	0.108996823		16.50505	47.98971	112.9294	89.58432	62.71572	64.97236
8	C21001	1327.319	3.108985	7.704174	10.07054	6.881356	6.479288	0.102399316		12.96219	30.36138	75.23658	98.34576	67.2012	63.27472
9	C21001	2750.856	11.46956	13.64102	16.82818	6.291857	5.213523	0.095902432		28.6839	119.5961	142.2386	175.4719	65.60685	54.36278
10	C21003	1714.282	5.751141	15.64529	18.9805	5.114118	5.548046	0.109086587		15.71488	52.72088	143.4208	173.9948	46.88127	50.85911
11	C21003	1182.536	1.789354	13.1568	9.575317	6.473819	6.450874	0.129065662		9.162284	13.8639	101.9388	74.1895	50.15911	49.98134
12	C21001	2515.127	12.33143	19.17631	13.62602	7.996813	8.466635	0.130971586		19.20361	94.15346	146.4158	104.038	61.05762	64.64482
13	C21001	913.1591	1.046892	14.19948	11.26744	6.748537	6.924194	0.101003692		9.040848	10.36489	140.5838	111.5547	66.81475	68.55387
14	C21002	2039.468	6.520071	17.46035	10.98923	5.944892	6.338406	0.134069014		15.21208	48.6322	130.2341	81.96699	44.34203	47.27719
15	C21003	1285.367	4.887828	10.64457	9.390903	5.728378	4.370251	0.104100801		12.34733	46.95284	102.2525	90.20971	55.02722	41.98095
mean		1571.112	5.13214	12.14312	11.64816	6.750588	5.864355	0.110305646		14.23137	46.56945	109.7834	107.1243	61.80414	53.24504

		pmol/brain								nmol/protein mg	pmol/protein mg				
Gyne	Colony	Tyr	DOPA	DA	NADA	TA	5HT	Protein mg		Tyr	DOPA	DA	NADA	TA	5HT
1	C21006	2385.554	9.028718	12.12036	14.42359	4.379112	4.909039	0.119887734		19.89823	75.30977	101.0975	120.3092	36.52678	40.94697
2	C21006	2617.636	11.71592	15.38252	14.23803	7.428595	6.290966	0.135275689		19.35038	86.60771	113.7124	105.252	54.91449	46.50478
3	C21006	1466.043	5.519702	11.39311	14.30301	5.788638	5.072006	0.113788869		12.88388	48.50828	100.125	125.6978	50.87174	44.57383
4	C21006	2989.441	10.38384	15.3059	15.3851	7.541366	5.944294	0.131773214		22.68625	78.80082	116.1534	116.7544	57.22988	45.11003
5	C21006	5580.911	21.95772	20.44529	13.01227	5.038812	5.842429	0.120270423		46.40302	182.5696	169.9943	108.1918	41.89568	48.57743
mean		3007.917	11.72118	14.92944	14.2724	6.035305	5.611747	0.124199186		24.24435	94.35923	120.2165	115.241	48.28771	45.14261
