# Supplementary material for: Caste-specific storage of dopamine-related substances in the brains of four Polistes paper wasp species
Source: PLoS One. 2023 Jan 26;18(1):e0280881. doi: 10.1371/journal.pone.0280881 (PMC9879392; doi:10.1371/journal.pone.0280881)
Supplement: S4 Table — (PDF) [file pone.0280881.s005.pdf]

S4 Table. Data of monoamine levels in the brain of *Polistes rothneyi* .

		pmol/brain								nmol/protein mg	pmol/protein mg				
Worker	Colony	Tyr	DOPA	DA	NADA	TA	5HT	Protein mg		Tyr	DOPA	DA	NADA	TA	5HT
1	R20022	8528.048	16.60906	15.14244	28.66699	8.826285	8.705956	0.130275073		65.46186	127.4922	116.2344	220.0497	67.75114	66.82749
2	R20025	6812.229	8.396803	18.38861	47.7251	8.493427	8.781676	0.142234751		47.89427	59.03482	129.2835	335.5375	59.71415	61.74072
3	R20002	7120.931	12.35189	17.74997	48.08219	6.578618	8.567953	0.161028532		44.22155	76.70619	110.2287	298.5942	40.85374	53.20767
4	R20005	12807.25	23.56522	22.79332	41.91741	9.385498	13.59304	0.233213737		54.91636	101.0456	97.73574	179.7382	40.24419	58.28577
5	R20002	7035.442	14.76671	19.61775	40.77084	4.606221	9.908142	0.154621562		45.50104	95.50228	126.8759	263.6815	29.79029	64.07995
6	R20002	6239.469	12.08791	23.04272	42.55644	4.396042	10.47783	0.146933197		42.46466	82.26805	156.8244	289.6312	29.91864	71.31013
7	R20005	10067.09	20.38141	25.6809	51.79375	6.646437	13.18434	0.15718435		64.04638	129.6657	163.3808	329.5096	42.28434	83.87819
8	R20025	7785.424	16.34829	22.67581	55.5406	4.06903	10.02021	0.161028532		48.3481	101.5242	140.8186	344.9115	25.269	62.22631
mean		8299.485	15.56341	20.63644	44.63166	6.625195	10.40489	0.160814967		51.60678	96.65487	130.1728	282.7067	41.97819	65.19453

		pmol/brain								nmol/protein mg	pmol/protein mg				
Gyne	Colony	Tyr	DOPA	DA	NADA	TA	5HT	Protein mg		Tyr	DOPA	DA	NADA	TA	5HT
1	R20001	25857.35	56.37103	50.5892	78.28855	18.43117	7.247715	0.24762523		104.4213	227.6466	204.2974	316.1574	74.43172	29.26889
2	R20001	20167.87	38.3076	18.45655	71.42615	6.117822	6.576237	0.244897754		82.35219	156.4228	75.36431	291.657	24.98113	26.85299
3	R20001	25056	38.97914	27.21762	69.948	10.29441	7.506406	0.22216071		112.7832	175.4547	122.5132	314.8531	46.33767	33.78818
4	R20001	21306.09	38.20452	26.00899	70.12382	9.890686	7.721332	0.262719546		81.09825	145.4194	98.99906	266.9151	37.64732	29.39002
5	R20001	28452.32	49.45951	39.24615	66.04638	8.310172	7.655639	0.23823636		119.429	207.6069	164.7362	277.2305	34.88205	32.13464
6	R20001	24849.58	51.02476	18.20031	61.88901	6.997262	5.500321	0.2189453		113.4967	233.048	83.12721	282.6688	31.95895	25.1219
7	R20002	14862.72	25.48236	20.37263	75.27389	6.875154	5.84246	0.222535508		66.78808	114.5092	91.54776	338.2556	30.89464	26.25406
8	R20001	12723.56	30.05801	37.86426	107.1195	5.77359	8.431208	0.273387363		46.54041	109.9466	138.5004	391.8231	21.11872	30.83979
9	R20001	22697.25	53.87804	18.48619	60.81125	6.25933	4.502669	0.226997596		99.98894	237.3507	81.43783	267.8938	27.57443	19.83576
10	R20001	16241.9	34.39247	23.05221	64.42604	6.187013	5.926871	0.301220125		53.92038	114.1772	76.52944	213.8836	20.53984	19.67621
11	R20001	25343.97	79.04514	24.84314	98.33876	8.61485	5.750296	0.357980171		70.79713	220.8087	69.3981	274.7045	24.06516	16.06317
mean		21596.24	45.01842	27.66702	74.88103	8.52286	6.605559	0.256064151		86.51051	176.581	109.6774	294.1857	34.03924	26.29324

Foundress		pmol/brain								nmol/protein mg	pmol/protein mg				
	Nest	Tyr	DOPA	DA	NADA	TA	5HT	Protein mg		Tyr	DOPA	DA	NADA	TA	5HT
1	R21F001	1360.175	1.445903	96.30336	144.8604	3.909894	35.65435	0.269847888		5.040526	5.358213	356.8802	536.8225	14.48925	132.1276
2	R21F002	1321.266	3.720354	282.8333	117.986	7.322489	29.32201	0.270427425		4.885842	13.75731	1045.875	436.2944	27.07747	108.4284
3	R21F003	2492.971	16.54035	403.8742	152.9854	5.625535	52.52967	0.306478313		8.13425	53.96907	1317.791	499.1719	18.35541	171.3977
4	R21F004	1796.663	4.747546	201.5158	146.7938	7.835495	33.16831	0.270653		6.638254	17.54108	744.5542	542.369	28.95034	122.5492
5	R22F001	1180.556	2.964321	168.9758	129.5197	6.416466	24.20414	0.382063025		3.089951	7.758722	442.272	339.0009	16.79426	63.35116
6	R22F002	2407.548	5.822381	276.0866	176.1794	10.92857	36.23145	0.42181051		5.707654	13.80331	654.5276	417.6742	25.90871	85.89508
7	R22F003	1829.685	8.544553	160.5464	141.1474	9.38116	21.73832	0.47082418		3.886131	18.14807	340.9902	299.7879	19.92498	46.17078
8	R22F004	1806.007	7.546143	177.5647	97.77008	6.065089	14.97905	0.411816063		4.38547	18.32406	431.1747	237.412	14.72766	36.37316
9	R22F005	1559.028	2.440334	179.0313	127.3961	12.2238	22.59986	0.421063572		3.702595	5.795643	425.1884	302.5579	29.03076	53.67327
10	R22F006	1867.596	3.558173	140.2948	140.9073	6.78807	30.55361	0.476573529		3.918798	7.466157	294.3822	295.6675	14.24349	64.11101
mean		1762.149	5.733006	208.7026	137.5546	7.649656	30.09808	0.37015575		4.938947	16.19216	605.3635	390.6758	20.95023	88.40773
