# Supplementary material for: Caste-specific storage of dopamine-related substances in the brains of four Polistes paper wasp species
Source: PLoS One. 2023 Jan 26;18(1):e0280881. doi: 10.1371/journal.pone.0280881 (PMC9879392; doi:10.1371/journal.pone.0280881)
Supplement: S5 Table — (PDF) [file pone.0280881.s006.pdf]

S5 Table. Data of monoamine levels in the brain of *Polistes jokahamae* .

		pmol/brain								nmol/protein mg	pmol/protein mg				
Worker	Colony	Tyr	DOPA	DA	NADA	TA	5HT	Protein mg		Tyr	DOPA	DA	NADA	TA	5HT
1	J21006	6930.077	37.31207	23.17598	8.790297	9.775651	12.41248	0.167622276		41.34341	222.5961	138.2631	52.4411	58.31952	74.0503
2	J21006	5681	22.01515	15.39302	20.03468	7.787428	9.07005	0.167713137		33.87332	131.2667	91.78182	119.458	46.43302	54.08073
3	J21006	5627.778	36.17963	19.59933	15.4223	5.80051	10.93054	0.175597191		32.04936	206.0377	111.6153	87.82773	33.03305	62.24779
4	J21006	4221.825	16.81682	21.77185	16.24121	10.08802	10.75355	0.156746127		26.93416	107.287	138.8988	103.6148	64.35899	68.60489
5	J21008	3403.905	16.34334	23.80239	14.01505	9.773655	11.3393	0.148239607		22.96218	110.2495	160.567	94.5432	65.93147	76.49302
6	J21008	3757.802	19.58281	20.01653	10.71221	7.273725	10.63392	0.150528424		24.96407	130.0938	132.9751	71.16406	48.32127	70.64395
7	J21006	2527.512	16.0534	17.40085	17.72732	6.462337	9.378345	0.154465813		16.36292	103.9285	112.6518	114.7654	41.83668	60.7147
8	J21006	3721.683	18.70181	12.55827	19.38927	7.462383	7.258099	0.142119722		26.18696	131.592	88.36401	136.4291	52.50773	51.07031
9	J21008	6563.046	25.78004	15.64669	12.34173	6.052462	8.189316	0.144927776		45.28494	177.882	107.962	85.15782	41.76192	56.50618
10	J21006	4606.2	16.97139	20.38224	14.58634	7.571453	8.956271	0.141295436		32.59978	120.1128	144.2527	103.2329	53.58597	63.38684
mean		4704.083	22.57565	18.97471	14.92604	7.804763	9.892187	0.154925551		30.25611	144.1046	122.7331	96.86341	50.60896	63.77987

		pmol/brain								nmol/protein mg	pmol/protein mg				
Gyne	Colony	Tyr	DOPA	DA	NADA	TA	5HT	Protein mg		Tyr	DOPA	DA	NADA	TA	5HT
1	J21005	5781.452	32.99738	26.01247	19.17928	9.10551	12.44166	0.178701791		32.35251	184.6505	145.5636	107.3256	50.95366	69.62245
2	J21010	2692.655	11.49807	28.18991	21.79337	10.30796	15.09648	0.196841074		13.67933	58.41294	143.2115	110.7156	52.3669	76.69375
3	J21005	5315.756	27.95248	25.70518	21.22274	11.01804	12.65793	0.215802324		24.63252	129.5282	119.1145	98.34344	51.05615	58.65523
4	J21014	5499.622	30.57761	23.60418	19.85093	6.798585	10.0021	0.151464442		36.30966	201.8798	155.8397	131.06	44.88568	66.03597
5	J21014	5128.786	34.03727	26.02987	19.76479	7.21973	11.16617	0.163268879		31.41313	208.4737	159.4295	121.0567	44.21988	68.39131
6	J21014	5443.093	25.75536	15.80082	23.61349	6.429937	7.346527	0.167531415		32.48998	153.7345	94.31559	140.9496	38.38048	43.85164
7	J21014	5396.491	32.51031	19.67163	30.03424	10.09573	9.251847	0.157675961		34.2252	206.1843	124.7599	190.4808	64.02837	58.67633
8	J21014	4686.947	28.99119	24.04294	18.52357	7.869431	10.2623	0.161088702		29.09544	179.9704	149.2528	114.9898	48.85154	63.70587
9	J21014	7879.004	40.86319	25.50153	22.02328	8.563858	9.909206	0.161316031		48.84204	253.3114	158.0843	136.5226	53.08746	61.42728
10	J21014	6058.289	24.02878	24.83678	27.3445	8.052713	11.77014	0.180781058		33.51175	132.9165	137.386	151.2575	44.54401	65.10718
11	J21014	5970.541	34.26339	24.0812	27.29349	6.883917	9.316985	0.171355526		34.843	199.955	140.5336	159.2799	40.1733	54.37225
mean		5441.149	29.40682	23.95241	22.78579	8.395037	10.8383	0.173257019		31.94496	173.547	138.8628	132.9074	48.4134	62.41266
