# Supplementary material for: Caste-specific storage of dopamine-related substances in the brains of four Polistes paper wasp species
Source: PLoS One. 2023 Jan 26;18(1):e0280881. doi: 10.1371/journal.pone.0280881 (PMC9879392; doi:10.1371/journal.pone.0280881)
Supplement: S6 Table — (PDF) [file pone.0280881.s007.pdf]

S6 Table. Tests of normality and equal variance in each group

	Shapiro-wilk-test		F-test
	Normality		Equal variance
**Head width**	Woker	Gyne	
P. snelleni	○	×	○
P. chinensis	×	○	×
P. rothneyi	○	○	×
P. jokahamae	×	○	×
**Lipid**			
P. snelleni	○	○	○
P. chinensis	×	×	○
P. rothneyi	○	○	○
P. jokahamae	○	○	○
**Tyrosine**			
P. snelleni	○	○	○
P. chinensis	○	○	×
P. rothneyi	○	○	×
P. jokahamae	○	○	○
**DOPA**			
P. snelleni	○	○	×
P. chinensis	×	○	○
P. rothneyi	○	○	○
P. jokahamae	×	○	○
**Dopamine**			
P. snelleni	○	○	○
P. chinensis	○	×	○
P. rothneyi	○	×	○
P. jokahamae	○	○	○
***N*-acetyldopamine**			
P. snelleni	×	○	○
P. chinensis	×	○	×
P. rothneyi	○	○	○
P. jokahamae	○	○	○
**Tyramine**			
P. snelleni	○	○	○
P. chinensis	○	○	○
P. rothneyi	○	×	○
P. jokahamae	○	○	○
**Serotonin**			
P. snelleni	○	×	×
P. chinensis	○	○	×
P. rothneyi	○	○	○
P. jokahamae	○	○	○

Significance level: $P = 0.05$
